# Malformation de la charnière cervico-occipitale et vitiligo

**DOI:** 10.11604/pamj.2018.30.146.15610

**Published:** 2018-06-20

**Authors:** Omar Boulahroud, Jalal El Benaye

**Affiliations:** 1 Departement of Neurosurgery, Military Hospital My Ismail, Meknes, Morocco; 2 Departement of Dermatology, Military Hospital My Ismail, Meknes, Morocco

**Keywords:** Vitiligo, chiari, mélanocyte, charnière cervico-occipitale, Vitiligo, chiari, melanocyte, cervico-occipital junction

## Abstract

Vitiligo is a relatively common multifactorial polygenetic dermatosis (0.5%-2% of general population) characterized by segmental or nonsegmental cutaneous depigmentation. Chiari malformation is a congenital cervico-occipital junction disease characterized by a migration of a part of the cerebellum through the foramen magnum.The association between these two pathologies is exceptional and poses a cause-effect problem. We here report the case of a 45-year old woman with no particular previous history who reported bilateral cervico-brachial neuralgias associated with thermal algesic dissociated sensory disorders lasting for 2 months. Cutaneous examination showed hypopigmented macular lesion on the neck and the posterior portion of the right forearm resistant to topical treatment based on strong corticosteroids (A). Brain MRI objectified Chiari malformation type II (B red arrow) with syringomyelia cavity at C6 (B white arrow). The patient underwent osteo-dural decompression of the posterior fossa with good neurological improvement and repigmentation of patches after 6 months. A few cases have been reported in the literature that show this association. Arguments in favor are: localization of lesions in dermatomes in correspondence with the malformation and regression after surgery. Etiopathogenesis of vitiligo is not clear, then several theories have been proposed to explain its occurrence;autoimmune theory, epidermal adhesion theory, biochemical theory and neuronal theory.This last seems to us to be the most likely explanation of this association. According to this theory the occurrence of skin lesions is due to the secretion of substances inducing melanocyte destruction by suffering neurons.

## Image en médecine

Le vitiligo est une dermatose relativement fréquente (0,5% - 2% de la population générale) d'origine polygénétique et de physiopathologie multifactorielle caractérisé par une dépigmentation cutanée pouvant êtres segmentaire ou non segmentaire. La malformation de Chiari est une maladie congénitale de la charniére cervico-occipitale qui consiste en une migration d'une partie du cervelet dans le trou occipital. Association de ces deux pathologies est exceptionnelle et pose une problématique de cause à effet. Notre cas concerne une femme de 45 ans sans antécédent particulier qui présente depuis 2 mois des névralgies cervico-brachiale bilaterale associé à des troubles sensitif de type dissociation thermo-algique. L'examen cutané trouve une lesion maculaire hypopigmentaire au niveau du cou et la face postérieure de l'avant bras droit résistante au traitement local à base de corticoides fort (A). L'IRM cerébrale objective une malformation de chiari type II (B flèche rouge) avec cavité syringomyélique en regard de C6 (B flèche blanche). La patiente a bénéficié d'une décompression ostéo-durale de la fosse cérébrale postérieure avec une bonne amélioration neurologique et repigmentation des plaques après 6 mois. Quelques cas dans la littérature témoignent de cette association. Les arguments en faveur sont: la localisation des lésions en regard des dermatomes concernés par la malformation et la notion de régression après chirurgie. L'étiopathogénie du vitiligo n'est pas claire et plusieurs théories ont été proposés pour expliquer son apparition; théorie autoimmune, théorie d'adhésion épidermique, théorie biochimique et théorie neuronale. Cette dernière nous parait la plus probable pour expliquer cette association dans notre cas elle suppose que l'apparition des lésions cutanées est due à la sécrétion au niveau des neurones soufrant de substances qui détruisent spécifiquement les mélanocytes.

**Figure 1:**
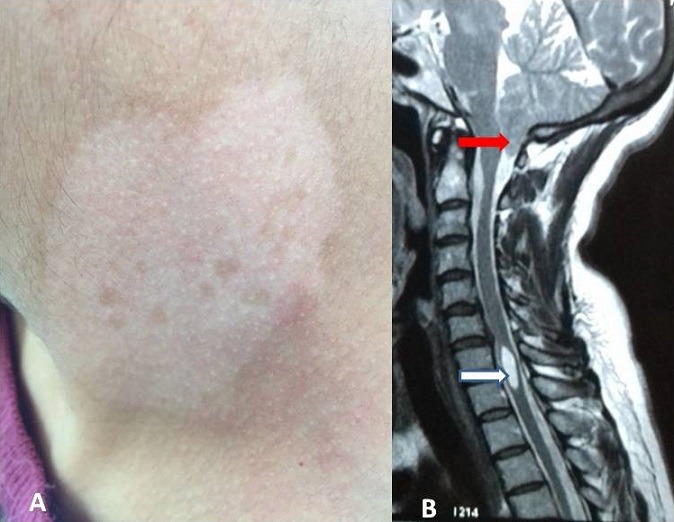
A gauche photographie montrant une lesion maculaire hypopigmentaire au niveau du cou (A); à droite IRM craniocervicale qui montre une malformation de chiari type II (B flèche rouge) avec cavité syringomyélique en regard de C6 (B flèche blanche)

